# What can clinicians do to improve outcomes across psychiatric treatments: a conceptual review of non-specific components

**DOI:** 10.1017/S2045796019000428

**Published:** 2019-08-15

**Authors:** S. Priebe, M. Conneely, R. McCabe, V. Bird

**Affiliations:** 1 Unit for Social and Community Psychiatry, WHO Collaborating Centre for Mental Health Service Development, Queen Mary University of London, London, UK; 2 School of Health Sciences, City University of London, London, UK

**Keywords:** Common factors, complex interventions, non-specific components, placebo, psychiatric treatment

## Abstract

**Aims:**

Psychiatric treatments have specific and non-specific components. The latter has been addressed in an extensive literature on the placebo-effect in pharmacology and on common factors in psychotherapy. In the practice of mental health care, pharmacological, psychotherapeutic and social treatments are combined in complex interventions. This paper aims to review non-specific components across diverse psychiatric treatments and consider implications for practice and research.

**Methods:**

We conducted a non-systematic review of non-specific components across psychiatric treatments, their impact on treatment processes and outcomes, and interventions to improve them.

**Results:**

The identified research is heterogeneous, both in design and quality. All non-specific components capture aspects of how clinicians communicate with patients. They are grouped into general verbal communication – focusing on initial contacts, empathy, clarity of communication, and detecting cues about unspoken concerns – non-verbal communication, the framing of treatments and decision-making. The evidence is stronger for the impact of these components on process measures – i.e. therapeutic relationship, treatment satisfaction and adherence than on clinical outcomes – i.e. symptoms and relapse. A small number of trials suggest that brief training courses and simple methods for structuring parts of clinical consultations can improve communication and subsequently clinical outcomes.

**Conclusions:**

Methodologically, rigorous research advancing current understandings of non-specific components may increase effectiveness across different treatments, potentially benefitting large numbers of patients. Brief training for clinicians and structuring clinical communication should be used more widely in practice.

## Introduction

Various treatments have been established in psychiatry, based on different ideas, approaches and methods. They are usually classified as biological, psychological and social, with each category containing a wide range of treatments. These treatments consist of specific and non-specific components. The specific components are defined by the theoretical model for how and why the given treatment is effective.

In addition to these specific components, there are also other components that may have a therapeutic effect. For example, the way treatments are presented to patients may fill them with optimism resulting in more positive engagement and improved mood (Thomas, 1987). The suggestion of improvement can raise expectations that then become self-fulfilling (Krell *et al*., 2004), and the respectful attention of clinicians may raise patients' self-esteem, and help them to overcome their distress (Robson, 1988). All these components that are not captured by the theoretical model but can still have a therapeutic effect are considered non-specific.

In psychiatry, there is a long history of considering non-specific treatment components, and the term ‘non-specific’ itself has occasionally been used in the literature since the 1960s (Honigfeld, 1964; Rickels, 1968). However, most of the psychiatric literature has used other terms. An extensive literature addresses non-specific components in psychopharmacology using the concept of placebo (Benedetti, 2008; Kirsch, 2014; Weimer *et al*., 2015), and in psychotherapy considering them as common factors across different schools (Huibers and Cuijpers, 2015; McAleavey and Castonguay, 2015; Wampold, 2015; Cuijpers *et al*., 2019). In contrast, there is little literature discussing non-specific components in social interventions.

In routine psychiatric care, pharmacological, psychological and social treatments are not delivered in isolation but are variably combined in complex interventions. This raises the question as to which components are non-specific across different treatments and how clinicians can utilise such components to improve outcomes across interventions, potentially benefitting large numbers of patients. We, therefore, conducted a review of non-specific components that have been shown to be associated with treatment uptake, satisfaction, adherence and outcomes across treatments and also reviewed the evidence for interventions to improve those components. The review focuses on what clinicians can do and say in treatment. It, therefore, uses the term treatment ‘components’. It avoids the frequently used term ‘factors’ which can imply treatment components, but also mediating processes and constructs about what may be going on in clinicians' and patients' minds such as attitudes, beliefs and experiences.

## Methods

We conducted a non-systematic review of the literature. A systematic search was not appropriate because a search term ‘non-specific’ would have been too restrictive and miss relevant literature that would not use the term, whilst including other search terms such as ‘placebo’ and ‘common factors’ across treatments would have yielded an unmanageable amount of literature. We, therefore, followed the approach suggested for conceptual reviews with (a) a wide search of disparate databases and sources, (b) forward and backward citation tracking, (c) safeguards against potential biases by using a team of researchers with different backgrounds and (d) some overlap of the searching, analysing and writing-up stages of the review (Lilford *et al*., 2001).

The synthesis was narrative and conducted in an iterative process by a team with a clinical-academic psychiatrist (SP) and three research psychologists at different career stages, educated in different countries.

## Results

All non-specific components identified in the review capture aspects of how clinicians communicate with patients. They fell into the groups of general verbal communication, non-verbal communication, treatment framing and decision-making. Research evidence is first presented for these components and then for interventions to improve clinical communication.

### General verbal communication

Extensive evidence shows that a more positive patient–clinician relationship is associated with better adherence and more favourable clinical outcomes across treatments (Fenton *et al*., 1997; Johansson and Jansson, 2010; Priebe *et al*., 2011*a*; McAleavey and Castonguay, 2015; Wampold, 2015; Berry *et al*., 2016; Green, 2017; Shattock *et al*., 2018; Strauss *et al*., 2018). Clinicians cannot directly control or vary the relationship, but they can influence it. The way to shape and change it is through communication. Communication can be very brief, as it is in an emergency, or occupy many hours, as in psychotherapy, and principles of good clinical communication in psychiatry have been suggested in the literature (Priebe *et al*., 2011*b*). Different clinicians achieve different treatment outcomes even if they prescribe the same medication (McKay *et al*., 2006) or provide the same type of psychotherapy (Crits-Christoph *et al*., 1991; Castonguay and Hill, 2017). Much of this variance in treatment outcomes is likely to be due to how they communicate with patients.

Good communication matters right from the very first contact. How psychiatrists introduce themselves can already make a difference. In an experimental study, patients preferred an introduction with an explanation about what to expect in the first consultation over brief introductions without explanation or longer introductions in which clinicians disclosed personal problems (Priebe *et al*., 2013). More research on the initial consultations has been conducted within primary care demonstrating the benefits of clear messages. In a randomised controlled trial (RCT) with patients with medically unexplained symptoms, a General Practitioner (GP) either gave a firm diagnosis with a positive prognosis or provided neither diagnosis nor prognosis (Thomas, 1987). Patients receiving the former message showed greater symptom improvement regardless of whether they received any treatment or not. In another RCT, patients with no definite diagnosis were randomly assigned to either a directive or a sharing style of communication. In the directive communication group, the GP made definitive statements about diagnosis, treatment, prognosis and follow-up. In the latter, the physician asked for the patient's opinion about the problem, treatment and diagnosis. Patients receiving a directive style of communication were more satisfied (Savage and Armstrong, 1990). In a similar patient group, the physician either provided a firm diagnostic label and prescribed medication, which was actually a placebo, or told the patient that there was no evidence of disease and that they did not require treatment (Thomas, 1978). Both groups were given clear, albeit very different, information about diagnosis and their prognosis and had similar outcomes. The two studies suggest that the content of some types of information may be less important than the way it is presented.

Beyond the initial consultation, a central component of beneficial clinical communication is empathy which concerns sensing patients' emotions and concerns and making them feel understood (Rickels *et al*., 1971; Elliott *et al*., 2018). A systematic review of the effect of empathy in healthcare consultations found that increased clinician empathy positively impacted on patients' pain, anxiety and satisfaction (Howick *et al*., 2018). Qualitative studies underline the importance of clinicians' empathy in psychiatry, and the related concept of positive regard has been shown to be linked with better outcomes in psychotherapy (Johansson and Eklund, 2003; Ljungberg *et al*., 2015; Ross and Watling, 2017).

A number of studies of video-recorded consultations have studied the empathy of psychiatrists in more detail and highlighted how they detect and respond to patients' hints about their concerns (Rimondini *et al*., 2006; Zimmermann *et al*., 2007; Del Piccolo *et al*., 2012; Xanthopoulou *et al*., 2018). Picking up on hints, as opposed to ignoring them or changing the topic, seems to strengthen the therapeutic relationship. Even the type of questions that clinicians use to elicit patient concerns appears relevant. Questions that propose an understanding of patients' experiences may be appreciated as a display of empathy and are linked with more positive relationships (Thompson *et al*., 2016).

### Non-verbal communication

Communication with patients is not solely verbal. Non-verbal behaviour, including posture, rate of speech, intonation and pitch of voice are critical in interpreting the meaning of verbal utterances and can convey additional messages. Non-verbal communication appears to be particularly relevant for showing that the clinician is listening, taking the patient seriously, demonstrating empathy and establishing a positive rapport (Beck *et al*., 2002).

Most research on non-verbal behaviour has been observational, exploring associations with patient satisfaction and treatment outcomes. One systematic review suggests that non-verbal indicators of clinician warmth and clinician listening are linked with greater patient satisfaction (Henry *et al*., 2012). Another study found that patients were more likely to attend their following appointment when their psychiatrists' tone of voice had been more positive (Cruz *et al*., 2013).

Communication, including non-verbal communication, is reciprocal. The non-verbal behaviour of psychiatrists and patients with schizophrenia during a consultation has been shown to be linked: when psychiatrists showed more pro-social behaviour in the form of gestures and open posture – inviting rather than avoiding interaction – patients reciprocated. This was associated with higher patient satisfaction and lower symptom levels (Lavelle *et al*., 2015). In psychotherapy, more co-ordination in patients' and clinicians' body movements, as assessed by automated analyses of videotapes, was associated with more positive therapeutic relationships and higher patient self-efficacy (Ramseyer and Tschacher, 2011). Thus, empathy can be rated in clinicians' speech and is also communicated in clinicians' non-verbal behaviour.

Non-verbal clinical communication has been investigated also in experimental designs. A study of actors pretending to be clinicians found that manipulating gaze and body orientation had a significant effect on how empathetic participants perceived their clinicians to be (Brugel *et al*., 2015). In progressive relaxation training for anxious women, therapists manipulated their voice volume, pitch and rate of speech. When the therapist decreased the tone, volume and rate of speech throughout the session, the patients were more relaxed (Knowlton and Larkin, 2006).

### Treatment presentation and framing

Patient expectation has consistently been linked to variation in clinical outcome across a range of medical disciplines, including psychiatry (Carver and Dunham, 1991; Safren *et al*., 1997; Mondloch *et al*., 2001). As with the therapeutic relationship, the beliefs and expectations of patients cannot be controlled by clinicians. However, they may be influenced by communication, especially by how treatment is presented (Glare *et al*., 2018). This is often referred to as framing and it can be manipulated in experimental research. A common way of framing treatment is for the clinician to tell a patient that the treatment has a 30% chance of success (gain frame) or alternatively that it has a 70% chance of failure (loss frame) (Levin *et al*., 2002; Moxey *et al*., 2003; O'Keefe and Jensen, 2007).

The majority of studies assessing the effect of treatment framing across different psychiatric conditions have focused on help-seeking behaviours and on the uptake of treatment. Findings from these studies have been mixed (O'Keefe and Jensen, 2007; Lueck, 2017) with more positive findings on treatment uptake than subsequent adherence (Mavandadi *et al*., 2017, 2018). Prospect theory may help explain this divergence (Tversky and Kahneman, 1979; Rothman *et al*., 2006). It suggests that individuals avoid risky behaviours when they are prompted to consider the potential gains. In contrast, individuals are more prepared to engage in risk-taking behaviour when prompted to consider possible loss. In terms of mental health care, it has been suggested that help-seeking is not risk-neutral (Lueck, 2018). Attending an initial appointment could result in a stigmatised diagnosis or in long-term treatment (Rothman *et al*., 2006). As a consequence, highlighting the potential losses associated with non-attendance by using a negative treatment frame may be more effective. In contrast, for individuals who do not perceive mental health treatment as risky, such as patients already in treatment, emphasising the benefits of behaviour through a positive treatment frame may be more effective.

In addition to presenting a specific positive or negative treatment frame as discussed above, clinicians can express their optimism or scepticism about treatment in more general terms. An experimental study using video-clips of real psychiatrists manipulated how optimistic or sceptical they were about a possible pharmacological or psychological treatment. Patients who were newly referred to mental health services preferred an optimistic treatment presentation. However, this was not the case for patients who had already been in psychiatric services for more than 2 years and had experienced that treatments in psychiatry are not always, at least not for them, a resounding and lasting success (Priebe *et al*., 2017*a*). Thus, the impact of clinician optimism or scepticism is likely to vary depending on patients' characteristics and experiences.

### Decision-making

Decision-making is central to most psychiatric treatment encounters, often relating to starting, reviewing or changing pharmacological or other treatments. Involving patients in the decision-making process is widely regarded as good clinical practice (NICE, 2011). The level of patient involvement depends mainly on clinicians' communication, as it requires informing patients, eliciting their preferences, discussing the pros and cons of different treatments and incorporating their preferences where possible into the decision (Edwards *et al*., 2010).

Much of the recent literature uses the concept of shared decision-making which suggests that decisions about treatment should be arrived at in a shared and non-directive discussion between patient and clinician (Hamann *et al*., 2003; Slade, 2017). More patient involvement in treatment decisions has been linked to symptom improvements and reduced substance misuse. This applies to patient groups with different diagnoses and in different settings, including primary care and inpatient treatment (Clever *et al*., 2006; Deegan and Drake, 2006; Hamann *et al*., 2006, 2017; Shay and Lafata, 2015; Perestelo-Perez *et al*., 2017). Systematic reviews found that patients with bipolar disorders want more involvement in treatment decisions, and more involvement is associated with better adherence, higher patient satisfaction and lower suicidal ideation (Fisher *et al*., 2016). In dementia, patients who were less involved in decisions about whether to start medication at the point of diagnosis were less satisfied than those who were more involved (Dooley *et al*., 2018).

Improving communication with involuntary patients may be particularly challenging (Thornicroft *et al*., 2013; Giacco *et al*., 2018*a*, 2018*b*). Research on this is limited but encouraging. Involving patients in treatment decisions and planning from the very first days of involuntary hospitalisation onwards was found to be feasible and valued by patients (Burn *et al*., 2019). An intervention combining components of shared decision-making with psychoeducation was reported to reduce re-hospitalisation rates in a RCT (Lay *et al*., 2017).

However, the precise preferences of patients for how decisions should be made can vary depending on patient characteristics, therapeutic situations – e.g. an acute emergency and a consultation in long-term treatments – and types of treatment (De Las Cuevas *et al*., 2013). For example, some patients want to be more involved in the decision-making process about psychosocial interventions than about which medication they are prescribed (Roter *et al*., 1997). To capture the variation of how patients want to be involved in decisions in a given situation, the OPTION scale has been developed (Elwyn *et al*., 2003). So far it has been applied more in general practice than in psychiatry, and where it has been used in psychiatry, patient involvement has been found to be very low regardless of their wishes (Goss *et al*., 2008). A related concept to involvement in decision-making is agreement about treatment. A review of effective clinician-patient communication in healthcare reported positive associations between patient-clinician agreement and patient outcomes (Stewart, 1995).

### Interventions to improve clinical communication

The importance of clinical communication raises the question of how clinicians' communication can be improved to make treatments more effective. Communication may be influenced through training or through interventions which structure communication during consultations, or both.

A Cochrane review of communication training in the context of severe mental illness in psychiatry identified only one RCT in this area (Papageorgiou *et al*., 2017). In a four-session training course, psychiatrists treating patients with psychosis practiced their communication skills with actors and listen to voices mimicking hallucinations on headphones whilst performing various tasks. The focus is on developing a shared understanding of symptoms, addressing positive and negative symptoms, empowering patients through agenda-setting and involving them in decision-making. The training led to improved observer-rated communication and more positive therapeutic relationships (McCabe *et al*., 2016).

In a primary care study, training in the form of structured discussions was tested to help clinicians elicit concerns from parents and children and to raise their treatment expectations. Three 1-hour discussions around video examples of family-clinician communication were followed by practice sessions with patients and self-evaluation. The training reduced the distress of parents and for some children, impairment across a range of disorders (Wissow *et al*., 2008). Another brief training focused on non-verbal behaviour. Clinicians recorded their own consultations in routine practice and reflected on three things they wanted to change in their non-verbal communication. Clinicians reported that the areas for improvement were apparent after watching fewer than five consultations. They focused on not interrupting the patient, attentive listening through feedback and looking at the patient rather than their medical notes. The training improved patient satisfaction and reduced distress (Little *et al*., 2015).

An alternative to training practitioners is to modify the structure of clinician-patient communication. Some interventions focus on improving decision-making through the use of decision aids. They are typically checklists assessing patients' preferences and providing information about available treatments. They are intended to help patients and clinicians arrive at a treatment decision. A Cochrane review of the effects of decision aids in people facing treatment decisions across medicine identified 105 studies covering over 31 000 participants (Stacey *et al*., 2017). Yet, the evidence for their effect on treatment outcomes in psychiatry is limited. Two cluster RCTs looking at the effect of decision aids aimed at improving shared decision-making with patients with depression and post-traumatic stress disorder found mixed results. Both studies failed to find effects on medication adherence or on symptom improvement, but the decision aid improved patients' treatment satisfaction and perceived involvement in decision-making (Mott *et al*., 2014; LeBlanc *et al*., 2015). An online decision aid informing young patients with depression about treatment options was tested in an RCT. It found higher treatment adherence and lower symptoms as compared to treatment as usual (Perestelo-Perez *et al*., 2017). Another similar online decision aid was assessed in a pre-post design. It was linked with improved knowledge of treatment options and less conflict with the clinician during the decision-making process (Simmons *et al*., 2017).

There are a small number of interventions to structure part of the patient-clinician meeting and directly guide communication. Focusing on 20 common needs, a communication checklist asks patients before a consultation to indicate the areas they want to discuss with their psychiatrist (Van Os *et al*., 2004). The checklist was found to improve the quality of patient-psychiatrist communication and induced changes in management immediately after the intervention. A more detailed method for structuring communication is DIALOG + (Priebe *et al*., 2015, 2017*b*), which is based on the quality of life research, concepts of patient-centred communication, and principles of solution-focused therapy. In their meetings with clinicians, patients rate their satisfaction with eight life domains and three treatment aspects, assisted by a graphical display on a tablet. Patients then decide which domain(s) to discuss in the given meeting. Each of the patient's concerns is then addressed in a four-step approach – understanding, looking forward, exploring options and finally agreeing on actions. In a cluster RCT, the use of DIALOG + regularly over a 6-months period improved outcomes and reduced treatment costs.

## Discussion

Although much less research in psychiatry has explored non-specific than specific treatment components, an increasing body of evidence highlights the importance of such components across treatments. The existing evidence suggests that the way clinicians generally communicate with patients both verbally and non-verbally, and how they frame treatments and involve patients in the decision-making process can influence uptake, adherence and outcomes of treatments. Overall, there is more evidence for the impact of non-specific components on process measures, such as satisfaction and adherence, than on clinical and social outcomes of treatments.

The summarising nature of our review did not consider the methodological quality of the referenced studies. While the review included some high-quality RCTs and meta-analyses, overall the studies are heterogeneous in their design and quality. Many studies were exploratory in nature and the results should be interpreted with caution. Moreover, we did not specify effect sizes. Most studies of non-specific components were intended to establish only that the given component is relevant in principle. Hardly any studies were designed and implemented with sufficient rigour to determine the effect sizes.

### Future research

Future research should go beyond establishing the effects of widely acknowledged non-specific components. There is no need for further studies showing that patients are more likely to come back for the next appointment if their clinician shows empathy and addresses their concerns. What is required is research that advances our understanding of non-specific components and the underlying mechanisms. This may include experimental studies with clinical and non-clinical samples that test the effects of varying such components in ways that would be feasible in clinical practice.

In RCTs, one might try to standardise the delivery of at least some non-specific components, in both the experimental and control groups. If achieved, this should reduce the variance in outcomes and therefore help to detect the effects of the specific treatment components being tested.

In addition, research should aim not only to understand non-specific components better but also to utilise them more effectively. The few studies on improving clinical communication indicate that such improvements are possible and can lead to better outcomes.

Much research on psychiatric treatments has focused on finding patient characteristics that predict a positive response to specific treatment methods. Recently, such efforts have sometimes been referred to as personalised medicine. For non-specific components, individual responsiveness may possibly vary even more than for specific ones. Which characteristics of the patient and context determine how best to communicate with the patient remains largely unknown. The literature provides some hints about patient characteristics and experiences predicting different responses to placebo or optimistic treatment framing (Bialik *et al*., 1995; Holmes *et al*., 2016). Yet, much more detailed research is required, and the categories often used in research, such as diagnoses, may not be very helpful for this.

### Implications for practice

Non-specific components are part of all treatments in practice, with one review suggesting that they explain up to 60% of the variance in outcomes (Walach *et al*., 2005). When clinicians communicate with patients it can always have an effect. This effect can be positive, but it can also be detrimental, which is sometimes referred to as a nocebo effect (Benedetti *et al*., 2007; Evers *et al*., 2018). Non-specific components should therefore not be ignored or devalued, but rather embraced and emphasised as a major part of what clinicians can do to help patients (McQueen and Smith, 2012).

Communication skills are important for all clinicians working in mental health care. Short and effective training courses exist and could be rolled out into routine care at limited costs. Another simple and even less expensive option for refining communication skills is video-recordings of consultations to review what works and what may be improved. Yet, neither training nor reviews of video-recordings are widely used in routine care.

Beyond that, a focus on communication skills may have positive implications for clinicians working in psychiatric care. Improving communication skills will require more training and supervision, but might also be an opportunity to strengthen the specific professional profile of clinicians in psychiatry. Clinicians in routine psychiatric care are expected to engage and communicate with patients in widely varying settings and treatment situations. These include acute crises, involuntary treatment or long-term rehabilitation, with variable time frames and changing treatment goals. The challenge for clinicians is to develop and flexibly utilise a repertoire of skills to achieve the optimal benefit for the patient. These skills are likely to vary across individuals and be influenced by personal styles and individual strengths so that much of the training will have to be individualised. Yet, such training may provide clinicians in psychiatry with a relatively unique skill-set and strengthen their professional expertise.

## Conclusions

Over the last four decades, extensive research on specific treatment components has led to only limited improvement of effects of psychiatric treatments. A stronger focus on non-specific treatment components in both practice and research may improve the effectiveness of complex interventions with pharmacological, psychological and/or social approaches. This would potentially benefit large numbers of patients across settings and treatment methods and therefore have a substantial public health effect beyond the improvement of confined specific treatment components.
